# Pain kept under wraps of myelin sheath

**DOI:** 10.3389/fpain.2025.1569515

**Published:** 2025-04-23

**Authors:** Veronica I. Shubayev

**Affiliations:** ^1^ Department of Anesthesiology, University of California, San Diego, La Jolla, CA, United States; ^2^ Research Service, VA San Diego Healthcare System, La Jolla, CA, United States

**Keywords:** myelin, pain, sex dimorphism, MBP, cholesterol, estrogen receptor, nuclear receptor

## Abstract

The myelin sheath serves both as insulator and metabolic powerhouse for large-diameter dorsal root ganglia (DRG) neurons—some of the longest cells in the body—transmitting sensory impulses from the periphery to the spinal cord. When myelin is damaged, bioactive fragments of myelin basic protein (MBP) are released, playing a pivotal role in pathological pain. MBP-derived peptides (MBPd) emerge as a ubiquitous yet sex-specific mediator of pain. In females, MBPd triggers a widespread transcriptional response across the peripheral nerve, DRG, and spinal cord, leading to persistent, treatment-resistant tactile allodynia—pain from normally innocuous touch. In contrast, males exhibit only a localized transcriptional response, confined to the nerve, which does not extend to the DRG or spinal cord or induce pain. The sex difference is driven by MBPd's interaction with lipids and regulation of nuclear receptor transcription factors, including the estrogen receptor (ESR) and the liver X receptor (LXR)/retinoid × receptor (RXR) complex—key regulators of lipid and cholesterol metabolisms mounting sex-dependent immunity. By unraveling these fundamental mechanisms of myelin remodeling, this work opens the door to innovative, non-addictive, personalized therapeutics and diagnostics for chronic pain.

## Pain, immunity, and biological sex

Pain caused by injury or disease of the nervous system (i.e., neuropathic pain), including treatment-refractory pain arising from normally painless tactile stimulus, such as from wearing clothing (mechanical allodynia), is among the leading causes of long-term disability (1–3). Compared to men, women are disproportionally affected both by chronic pain (4–9) and autoimmune (10) conditions, raising a question of common mechanisms. Indeed, pro-nociceptive effects of immunoglobulin (Ig)M/IgG autoantibodies contribute to persistent pain in arthritis (11), fibromyalgia (12), and complex regional pain syndrome (CRPS) (13)—all female-prevalent conditions. Although neuropathic pain in principle (e.g., caused by PNS trauma) is not inherently more prevalent in females, we have argued that autoimmune mechanisms selectively contribute to certain neuropathic pain phenotypes, at least in females. Why?

Rodent models of PNS trauma have revealed sex-dimorphic immune mechanisms maintaining mechanical allodynia, with innate immune cells (microglia, macrophages) prominent in males, and adaptive immune cells (B/T lymphocytes) — in females (14, 15, 31, 33). PNS trauma initiates Wallerian degeneration, a systematic process of axonal demyelination and degeneration, subsequent removal of cell and myelin debris, and eventually axonal regeneration and remyelination, first described by Augustus Waller in 1850. This process involves sequentially recruited hematogenous immune cells, including neutrophils (within hours), macrophages (days to weeks) and B/T lymphocytes (week(s) post-injury) (16–18). Previous insights into the immune regulation of pain, including pro-nociceptive mechanisms (e.g., T helper (Th)1/17 cells) and anti-nociceptive mechanisms (e.g., Th2/Treg cells), primarily derived from studies on male rodents, are now being refined through research involving both sexes. Our 2010 mass spectrometry analysis of female rat sciatic nerves identified *Antigen Presentation, CD28 Signaling in T-helper Cells*, and similarity to *Pathogenesis of Multiple Sclerosis* after sciatic nerve chronic constriction injury (CCI) (19). Because this type of PNS trauma represents “sterile” (i.e., pathogen-free) inflammation, we implicated myelin autoantigenic epitopes we had observed released in PNS trauma (20) in the development of neuropathic pain, as detailed below.

## Myelin autoantigens in pain

Myelin sheath enables rapid, saltatory propagation of touch, pressure, position, movement, and vibration afferent traffic to DRG thence the spinal cord (21). PNS injury induces loss of the structural and molecular integrity of myelin on Aβ/δ-type-afferent neurons, leading to neuropathic pain states. There are at least three distinct mechanisms of myelin involvement in neuropathic pain: (a) ectopic insertion of voltage gated ion (Nav) channel, which is typically segregated by myelin into the nodes of Ranvier (22–24); (b) release of pro-nociceptive lipid metabolites from myelin, a metabolic warehouse of lipids; and (c) release of immunodominant autoantigenic epitopes, including myelin basic protein (MBP) α-helix 87-VVHFF-91 region.

**Figure 1:**
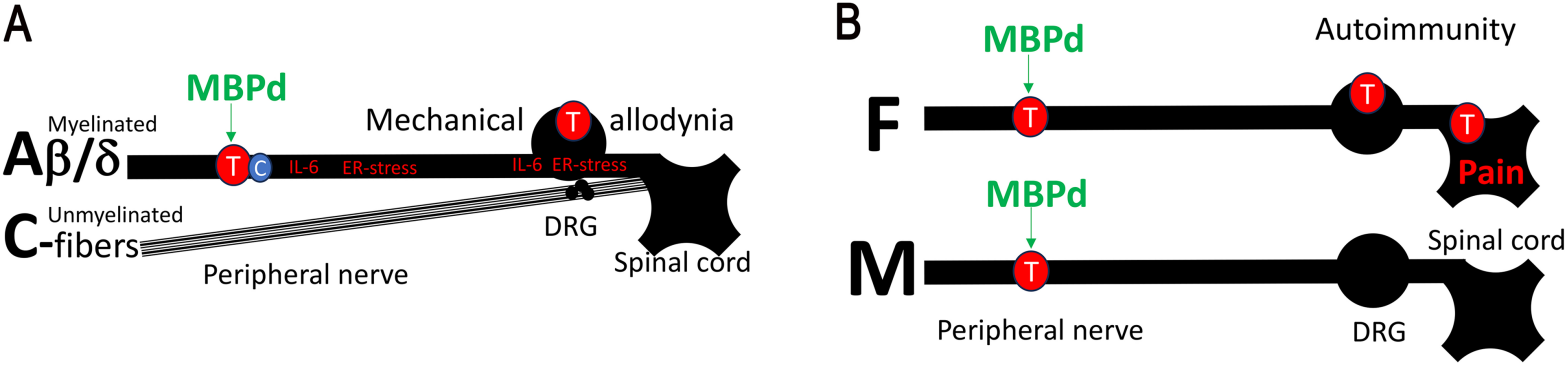
MBP-induced autoimmune mechanisms of neuropathic pain in female PNS. **(A)** Myelin autoantigens drive autoimmune remodeling of myelinated mechanosensory Aβ/d-afferents. Peripheral nerve injury causes myelin degradation and the release of pro-nociceptive, immunodominant MBP-derived epitopes (MBPd) normally sheltered from immunosurveillance. Exposure to MBPd induces IL-6 expression, ER-stress, and T cell activation at the injury site and the segmental DRG and spinal cord. Unmyelinated thermal/pain C-nociceptors are spared. As a result, intraneural injection of MBPd into an intact sciatic nerve induces T cell-dependent mechanical allodynia, without a change in thermal sensitivity. **(B)** IN MBPd triggers T/B cell activation in the nerves of both sexes. However, in females, T/B cell-related signaling progresses from the nerve to the DRG and spinal cord, whereas in males it remains localized to the nerves.

The immunodominant MBP87-91 epitope contributes to multiple sclerosis (MS), an autoimmune demyelinating disorder (25, 26). Peptides comprising MBP87-91 (MBPd) induce MS/experimental autoimmune encephalomyelitis (EAE) after systemic (subcutaneous), adjuvant-assisted immunization (19, 20, 27–31). After PNS trauma, the same epitope is proteolytically released and presented to T cells via major histocompatibility complex (MHC)II-expressing Schwann cells and macrophages (20, 29). Local (intraneural), adjuvant-free injection of MBPd into an intact sciatic nerve (IN-MBPd) is sufficient to induce a robust, T cell-dependent mechanical allodynia sustained for several weeks, with no thermal/heat sensitivity or motor deficits (19, 20, 27–31). We have implicated selective autoimmune remodeling increasing A-afferents (e.g., tactile) input while sparing unmyelinated C-nociceptors (e.g., heat) (32) (Figure 1A).

MBPd effects in the PNS are sexually dimorphic. After equal dose IN injection in sciatic nerves of both sexes, MBPd induced pain-like behavior in female, not in male, mice (33). This finding correlated to prominent T cell activity in nerves of both sexes, albeit female-specific interleukin (IL)-22 and CD137 (41BB) activation, yet a striking difference in ipsilateral DRG and spinal cord, where B/T lymphocytes signaling was entirely female-specific (33) (Figure 1B). Further, MBPd is released in nerves of both sexes after PNS trauma (e.g., CCI) (19, 20, 27–31), anti-MBPd autoantibody is detected exclusively in serum of female, not male, rats with CCI (31). This female-specific engagement of IgM autoantibodies corresponds to female prevalence of B cell action post-CCI (31, 34). Serum anti-MBP autoantibody in female CCI rats (31) correlates with female-specific DRG and spinal cord B/T cell activity and IgM-related genes after IN MBPd (33) and suggests potentially sex-dependent anti-MBPd IgM immune complex deposition on the damaged myelinated afferent neurons.

## MBP, membrane phospholipids and ER-stress

**Figure 2:**
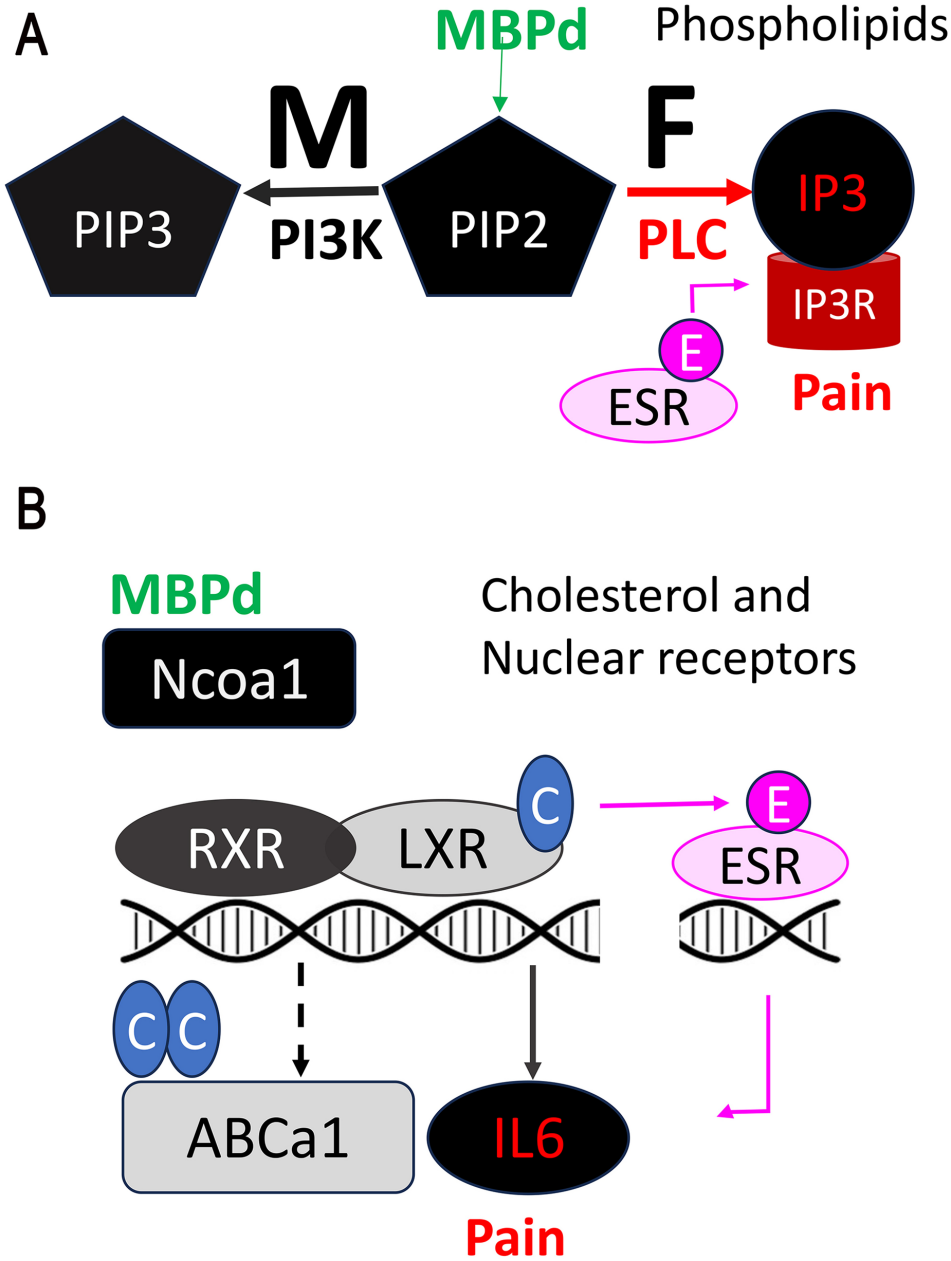
MBP-induced pain by regulation of lipid and cholesterol metabolism. **(A)** The release of MBPd drives sex-specific metabolism of the PIP2 phospholipid in peripheral nerves. In females, PIP2 is broken down into IP3 by phospholipase C (PLC), activating IP3R mediated ER stress and calcium-dependent pain signaling. Estrogen/ESR1 co-activates IP3R-dependent nociception. Mechanical allodynia in females caused by IN MBPd is reversed by IP3R blockade. In males, PIP2 is converted into PIP3 through phosphatidylinositol 3-kinase (PI3K) activity, without activation of IP3R-induced ER stress or pain-like behaviors. The PI3K and PLC/IP3R activity respectively persist to male and female spinal cord after IN MBPd. **(B)** Upon release, MBPd inhibits cholesterol efflux by suppressing LXR/RXR expression. By Ncoa1 binding and sequestration, MBPd also prevents transcriptional activity of LXR/RXR. MBPd promotes cholesterol synthesis by activation of a cholesterol reductase DHCR7. LXR stimulation suppresses Estrogen/ESR1-induced IL-6 activation in DRG neurons and attenuates IN MBPd-induced mechanical allodynia.

During myelin compaction, the cationic MBP (isoelectric point of >11) binds apposing membranes via electrostatic interactions with anionic lipids, such as phosphatidylinositol 4,5–bisphosphate (PIP2) (25, 35). Thus, IN MBPd stimulated PIP2 metabolism in sciatic nerve, yet with intriguing sex differences (Figure 2A): PIP2 hydrolysis to inositol triphosphate (IP3) via phospholipase C (PLC) induction was selectively observed in female nerves. Male nerves exposed to IN MBPd displayed preferential PIP2 phosphorylation to phosphatidylinositol 3,4,5–bisphosphate (PIP3) by stimulation of phosphoinositide 3–kinase (PI3K) activity (33). The mechanism also advanced to DRGs and spinal cords in a sex-dependent manner. In females after IN MBPd, IP3 receptor (IP3R) induction on endoplasmic reticulum (ER), along with ESR1, were predicted to activate Ca^2+^-mediated ER-stress, voltage-gated Ca (Cacna)2d1 and mechanical allodynia, mitigated by IT administration of IP3R inhibitor (33).

## MBP, cholesterol and nuclear receptors

Lipid energy expenditure in cells of the PNS is regulated via a hierarchical nuclear receptor (NR) transcription factor network that includes estrogen receptor (ESR), androgen receptor (AR), liver × receptor (LXR), retinoid × receptor (RXR), vitamin D receptor (VDR), thyroid hormone (TH) receptor, progesterone receptor (PR) and peroxisome proliferator-activated receptor (PPAR) subfamilies. Pro- and anti-nociceptive action of NRs may relate to sex differences in ligand levels, including sex hormones (e.g., estrogen, testosterone) and per our recent findings, cholesterol precursors (desmosterol and 7-dehydrocholesterol, 7-DHC) and metabolites (e.g., oxidized cholesterol (oxysterol) 25-OHC) (19, 36).

According to our transcriptomic-based prediction, the pro-nociceptive effect of IN MBPd related to *female-specific cholesterol accumulation* in the nerve (33) via three mechanisms: (a) control of NR expression. Like known exogenous toxins (37), MBPd activates ESR1/Ca^2+^-dependent ER-stress in female DRG and spinal cord (24). MBPd also downregulates LXRa and RXRa, which act as obligate heterodimers activated by oxysterols to induce ATP binding cassette (Abc)-mediated cholesterol efflux and repress IL-6-mediated neuroinflammation and mechanical allodynia, reversed by IT administration of LXR agonist or IL-6 inhibitor (24); (b) control of NR ligand synthesis; e.g., by female-selective induction of 7-dehydro cholesterol reductase (DHCR7), MBPd is expected to convert 7-DHC to cholesterol selectively in female nerve (19, 36); (c) control of NR co-activators and co-repressor activity; by binding, and presumably sequestering nuclear receptor co-activator (Ncoa)1, also known as steroid receptor coactivator (Src)1 in injured nerves of both sexes (24), MBPd may regulate several Ncoa1/Src1-dependent transcription factors, including ESR, AR, LXR, RXR, VDR, PPAR, as well as C-Fos, C-jun, cyclin D1, and STAT3 (38, 39). That MBPd's effects on Ncoa1 binding, LXRa/RXRa expression are comparable between sexes suggests that a complex co-regulation is at play (Figure 2B). In female and male DRG neurons, LXR activation repressed ESR1-stimulated IL-6 (24). Given comparable ESR1 levels in male and female DRGs (36), high circulating estrogen levels in females, local synthesis of estrogens, sex-dependent control of NR ligands, noted above, and trans-regulation of NR systems, such as partial antagonism of ESR1 by 25H7 oxysterol (40), contribute to sex-dimorphic mechanisms of pain.

## Other MBP functions in the PNS

MBP is an intrinsically unstructured protein with multiple binding partners, such as tubulin, actin, Ca-calmodulin (25, 35). In addition to Ncoa1 (36), we observed sex-dependent interaction with Cacna, kinases and phosphatases. In females exposed to IN MBPd, higher *vesicular formation* in the nerve associated with pain phenotype (33). MBPd promotes *nuclear translocation of* cyclin-dependent ATPase-kinase (CDK) family members, expressed sex-specifically in the PNS (25, 26, 33). MBPd mutagenesis at H89G stimulates ATPase activity of CDK5 (28). In DRG neurons, MBPd binds ATP synthase and voltage-dependent anion-selective channel-1 (VDAC-1) and a functional anchor to cytoskeletal proteins. It is important to note that MBPd lacks the ability of the full-length MBP to interact with α-tubulin or cell-surface scavenger receptor low-density lipoprotein receptor related protein 1 (LRP1/CD99), thus escaping LRP-1-mediated endocytosis (41).

## MBP in molecular mimicry

The invariant 87-VVHFF-91 motif of MBPd is evolutionarily conserved (homologous across mammals, amphibians, fish). Its structural similarity with T cell epitopes of Influenza A and Epstein Bar Virus (EBV), the most common human virus, has led us to suggest that MBPd-based molecular mimicry contributes to neuropathic pain associated with viral neuropathies and idiopathic neuropathic pain states (41). Our findings of structural similarity between this MBPd motif and the p65-like protein of *common cold human coronavirus (HCoV) OC43* suggests a related mechanism of neuropathic pain (42). In addition to viral polypeptides, we have reported structural homology of this MBPd motif with muscarinic *acetylcholine (Ach) M2 receptor* (cytosolic motif), an inhibitory G-protein-coupled receptor on sensory neuron and a key epitope in CRPS (43). MBPd may exert its effects, at least partially, by counteracting the pain-inhibitory downstream signaling associated with Ach M2 receptor activity.

## Clinical implications

More than 600 million people worldwide suffer from chronic pain, making it the leading reason patients seek medical care. Just in the U.S. alone, the impact is significant, with an estimated annual economic cost of $650 billion and an increasing number of opioid overdose deaths (1–3). In response to this staggering impact, about seven years ago (April of 2018), the National Institutes of Health (NIH) developed the Helping to End Addiction Long-term (HEAL) Initiative aiming to provide scientific solutions to the opioid crisis and discovery of both reliable biomarkers and novel, non-addictive alternatives to treat and prevent pain. The pioneering efforts of my team to characterize MBPd as a mediator of pain has been supported since 2012, by a preceding initiative, NIH R01 Blueprint Grand Challenge on Chronic Pain, put forth by 25 NIH institutes and centers to “*recognize innovative research to identify novel targetable mechanisms of pain”*. Our work has brought both conceptional innovation to our understanding of treatment-refractory neuropathic pain states, and opportunities for novel biomarker developments and non-addictive therapeutic alternatives.

Our findings suggest that myelin remodeling and subsequent MBPd release drive persistent pain associated with various pathological conditions, including:

(a) Peripheral nerve injuries caused by trauma, metabolic diseases like diabetes, drugs, and toxins.
(b) Painful autoimmune demyelinating disorders, such as multiple sclerosis, Guillain-Barré syndrome, and chronic inflammatory demyelinating polyneuropathy, which release MBPd.
(c) CRPS and other pain syndromes involving molecular mimicry between MBPd and the Ach M2 receptor.
(d) Idiopathic neuropathic pain potentially triggered by viruses like common cold HCoV-OC43 through molecular mimicry with MBPd.

Both MBPd and MBPd-reactive autoantibodies show promise as biomarkers (31), which we have begun investigating clinically in MS pain, fibromyalgia, and low back radiculopathy (20, 44).

The targeted release of MBPd in nerves—both before demyelination and after myelin repair (19, 31)—supports its role in idiopathic pain without clear neuropathological findings.

Therapeutically, a cyclized head-to-tail altered peptide ligand (APL) double Ala 91,96 mutant of MBP87-99 presents a low-cost, non-addictive immunomodulatory neurotherapy, effective in treating experimental paralytic EAE disease, spinal cord trauma (45–50), and neuropathic pain (51). MBPd-dependent pain responds to systemic gabapentin and intrathecal interventions targeting neuroinflammation and ER-stress—such as IL-6 neutralization, IP3R blockade, and LXR stimulation—whereas lidocaine, ketorolac, and NMDA receptor antagonist have shown no efficacy (27, 33, 36).

## Data Availability

The original contributions presented in the study are included in the article/Supplementary Material, further inquiries can be directed to the corresponding author.
